# Examining the functional space of gut microbiome‐derived peptides

**DOI:** 10.1002/mbo3.1393

**Published:** 2023-12-12

**Authors:** Ying‐Chiang J. Lee

**Affiliations:** ^1^ Department of Molecular Biology Princeton University Princeton New Jersey USA

**Keywords:** anticancer, antimicrobial, bioactivity, machine learning, microbiome, peptide

## Abstract

The human gut microbiome contains thousands of small, novel peptides that could play a role in microbe–microbe and host–microbe interactions, contributing to human health and disease. Although these peptides have not yet been systematically characterized, computational tools can be used to elucidate the bioactivities they may have. This article proposes probing the functional space of gut microbiome‐derived peptides (MDPs) using in silico approaches for three bioactivities: antimicrobial, anticancer, and nucleomodulins. Machine learning programs that support peptide and protein queries are provided for each bioactivity. Considering the biases of an activity‐centric approach, activity‐agnostic tools using structural and chemical similarity and target prediction are also described. Gut MDPs represent a vast functional space that can not only contribute to our understanding of microbiome interactions but potentially even serve as a source of life‐changing therapeutics.

## Graphical abstract

The human gut microbiome comprises thousands of small, novel peptides that could potentially contribute to microbe‐microbe and host–microbe interactions, thereby affecting human health and disease. This article proposes exploring the functional space of gut microbiome‐derived peptides using in silico approaches that include machine learning tools, as well as activity‐agnostic approaches.

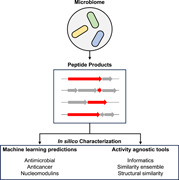

## 1 INTRODUCTION

The gut is a densely populated space where microbes and host cells interact with each other. This community, called the microbiome, is diverse and abundant. In humans, the gut microbiome is estimated to consist of several 100 bacterial species along with other microscopic eukaryotes and a larger number of viruses (Matijašić et al., 2020; Qin et al., 2010). In sum, the gut microbiome has an enormous biosynthetic potential that also contributes to its metabolic, physiological, and regulatory effects on the host or other microbes. The microbial effectors can vary in size, from small molecules to multidomain proteins that play a role in the microbiome's ecology and human health. Understanding how the gut microbiome contributes to health and disease has been of particular interest recently, and these compounds can shed light on this dynamic and hopefully improve human health (Cohen, 2015; Hou et al., 2022; Valdes et al., 2018; Vijay & Valdes, 2022). However, these same compounds could also be used for therapeutic applications that extend beyond their natural role in microbe–microbe and host–microbe interactions. Thus, the gut microbiome can be a rich source of biologically relevant products.

These products can come in the form of small molecules, peptides, and proteins. Recent studies using single‐strain cultures, community cultivation, sequencing, and omics approaches have shown that the gut microbiome encodes numerous small proteins and peptides (Petruschke et al., 2021; Sberro et al., 2019). Many of these peptides are predicted to be secreted. Other research groups have highlighted the role of gut microbiome‐derived peptides (MDPs) as antimicrobials and immune modulators (Fernández‐Tomé et al., 2019; Ma et al., 2022). Peptides can be the product of ribosomal and nonribosomal synthesis as well as the result of degradation products from larger proteins and other peptides. These peptides are sometimes referred to as cryptic peptides or cryptides. Functional peptides are an interesting class of molecules that span from only consisting of small di‐ and tripeptides that are more similar, to larger compounds of 50 amino acids or more with defined secondary and tertiary structures. These peptides can have extracellular or intracellular targets and impact both host and microbe physiology. Thus, research on gut MDPs could reveal interesting peptide effectors of microbe–microbe and host–microbe interactions and potentially lead to applications for the improvement of human health.

In this article, the functional space of gut MDPs is proposed to be probed using in silico approaches for three bioactivities: antimicrobial, anticancer, and nucleomodulins. Machine learning (ML) programs that support peptide and protein queries are supplied for each bioactivity. Considering the biases of an activity‐centric approach, activity‐agnostic tools for the characterization of MDPs are also described. Gut MDPs represent a vast functional space that can not only contribute to our understanding of microbiome interactions but potentially even serve as a source of life‐changing therapeutics.

## 2 BIOACTIVITIES OF INTEREST

### 2.1 Antimicrobial

The gut microbiome can be a source of novel antimicrobial peptides (AMPs). AMPs are typically short, positively charged peptides, often adopting a helical conformation, and causing membrane disruption that results in cell death or inactivation and inhibition across a broad range of microbes, including bacteria, viruses, and parasites. Several recent reviews cover AMPs and their targets as well as database repositories in more detail (Huan et al., 2020; Lazzaro et al., 2020). Antimicrobial MDPs will likely be found in two forms: either they are de facto AMPs or have incidental antimicrobial activity. In the former case, gut‐based AMPs can help carve out ecological niches and engage in microbial competition in the crowded gut microbiome. In the latter case, protein and peptide degradation products may have antimicrobial activity or segments of the genome could encode nonexpressed proteins and peptides that are antimicrobial. Independent of how an AMP originates, computational tools will be needed to systematically search the genomic and proteomic space for AMPs. A recent study using multiple natural language processing neural network models has identified gut‐specific MDPs with antimicrobial activity at positive prediction rates of over 83% from a candidate list of 216 peptides (Ma et al., 2022). Several short‐listed AMPs were potent against drug‐resistant Gram‐negative bacteria in vitro, and several peptides were found to be effective against *Klebsiella pneumoniae* lung infections in mice. Another study assembled a catalog of 863,498 peptides from metagenomic and genomic datasets that helped guide in vitro and in vivo assays demonstrating AMP activity against pathogens (Santos‐Júnior, 2023). These results highlight the potential for AMP discovery from the gut microbiome. Many publicly available tools using ML algorithms have been developed and will help mine the gut microbiome for potential AMPs. These include DRAMP 3.0, APD3, AI4AMP, iAMPpred, and AMP Scanner (Lin et al., 2021; Meher et al., 2017; Shi et al., 2021; Veltri et al., 2018; Wang et al., 2016).

### 2.2 Anticancer

While the gut microbiome is involved in cancer progression and treatment outcomes, less attention has been placed on examining the proteins encoded by the gut microbiome for anticancer activity (Li et al., 2021; Sivan et al., 2015; Zhao et al., 2023). However, recent work has shown that gut metagenomes can be mined for anticancer peptides (ACPs) (Ma et al., 2020). ACPs are in many ways similar to AMPs and can have multiple antitumor mechanisms (Xie et al., 2020). They are also short and can come in helical and cyclic conformations. ACPs can disrupt cell membrane integrity, induce apoptosis, and inhibit tumor angiogenesis. These peptides have been found in various environments, not just the gut microbiome, and serve as inspiration for gut peptide discovery efforts. Various reviews have shown how ACPs can be effective against different tumors and cancer cells (Jafari et al., 2022; Karami Fath et al., 2022; Li et al., 2023). In addition to direct inhibition of cancer cells, ACPs can also have an immunomodulatory effect as demonstrated in mouse models (Çuburu, 2022). The efficacy of immunotherapy in solid tumors has been demonstrated and peptide compounds could be an alternative to the larger and bulkier antibodies that are typically found in immunotherapies. To that end, small peptides have been characterized in various tumor models and show possible utility as checkpoint inhibitors (Liu et al., 2019; Yin et al., 2021). One peptide, CLP002, was discovered to block PD‐1/PD–L1 interactions, and it facilitates T‐cell survival and inhibits CT26 tumors in mice (Liu et al., 2019). Similar to how ML has been used to identify AMPs, more work is now being devoted to ACP prediction from sequence composition. Recent publications have shown how ML algorithms can support the differentiation of ACPs versus non‐ACPs and many web servers for ACP prediction are now online. These tools include xDeep‐AcPEP, MLACP 2.0, and ACPred‐BMF (Chen et al., 2021; Han et al., 2022; Thi Phan et al., 2022).

### 2.3 Nucleomodulins

Movement of macromolecules over 40 kDa into the cell nucleus typically requires a short signal peptide called the nuclear localization sequence (NLS) (Keminer & Peters, 1999; Lu et al., 2021). The NLS is recognized by importin proteins and the entire complex is transported through the nuclear pore from the cytoplasm into the nucleus. Once inside, the tagged cargo is released from its transport protein complex and can regulate gene expression. While NLSs are expected in eukaryotic organisms, their presence in bacteria is more surprising. Studies have revealed that bacterial proteins with NLSs can play a role in host–microbe interactions and promote bacterial survival (Ma et al., 2020; Pourpre et al., 2022). Despite this, more research is needed into the origin and function of bacterial NLSs. Did bacteria independently evolve NLS to target the eukaryotic nucleus? Did the NLSs already exist before eukaryotes arose? What genes are often affected by NLS‐containing bacterial peptides and proteins? Further investigation into the presence of NLSs in gut MDPs could reveal how microbes modulate host gene expression and contribute to health and disease. ML tools, such as NucPred, NLSdb, NLStradamus, and SeqNLS have been developed to predict and classify whether a peptide or protein query contains NLSs (Bernhofer et al., 2018; Brameier et al., 2007; Lin & Hu, 2013; Nguyen Ba et al., 2009).

## 3 ACTIVITY AGNOSTIC MDP CHARACTERIZATION

The use of ML programs to mine the peptide space for functionality is an activity‐aware approach. While this can be beneficial when searching datasets for how certain peptides with a defined function contribute to health or disease phenotypes, other methods are needed to characterize peptides that do not involve entering sequence queries into multiple ML programs or servers—although this method of brute forcing activity discovery could be fruitful. Thus, activity and sequence alignment agnostic tools could be effective in characterizing MDPs and rely on known molecular interactions between peptide ligands and protein receptors. Three such in silico methods include (1) a suite of tools from the Swiss Institute of Bioinformatics, (2) the similarity ensemble approach (SEA), and (3) the Dali server for structural similarity.

Both former sets of methods (1 and 2) allow for ligand‐based screening but have different outputs. Users of the Swiss Institute for Bioinformatics have the option of searching through multiple tools. SwissSimilarity can examine different drug libraries for targets that are molecularly similar to the search query (Bragina et al., 2022). This allows researchers to find not only similar compounds but also their corresponding receptors in an unbiased manner. To that end, the SwissTargetPrediction tool predicts potential macromolecular targets from three species for search compounds (Daina et al., 2019). The other bioinformatic method is SEA which groups proteins based on ligand similarity and can be used to search for ligand–protein targets (Keiser et al., 2007). A list of potential protein receptors for the searched compound is produced and can be grouped to provide a picture of the class of protein the peptide may interact with. These two in silico methods could be useful in the nonbiased characterization of novel gut MDPs and help lead to the design of new gut MDP‐based agonist and inhibitor compounds. The third method, examining structural similarity with the Dali server, also provides an activity‐agnostic approach to the functional characterization of peptides (Holm et al., 2023). As opposed to traditional sequence alignment, Dali examines three‐dimensional structures of the input to structures in the Protein Data Bank through either a heuristic, exhaustive, or hierarchical search. Outputs include various matching scores and a superposition viewer that can offer more spatial context. This approach can help viewers conceptualize and visualize how a gut MDP structure could relate to function.

## 4 CONCLUSION

Peptide function predictors that rely on ML algorithms can aid in the functional characterization of gut MDPs. These MDPs have various bioactivities, including antimicrobial, anticancer, and nucleomodulins, which can be relevant to the local gut microbial community and human health. Discovering novel MDPs and researching new functions for known MDPs can provide insights into microbe–microbe and host–microbe interactions and lead to the development of new peptide therapeutics. Other applications of ML algorithms and research can expand the bioactivities of the gut microbiome to include quorum sensing, protease inhibition, and even antioxidant MDPs.

Despite the potential of ML in predicting function from sequences, several challenges exist that may hinder their use. The quality of the training data is critical in developing an ML approach. Training datasets that contain incorrectly entered peptide sequences or incorrect activity annotations can affect the quality of the program. Training data should also be appropriately sized according to the number of parameters. Advances in structural biology and modeling, such as AlphaFold, can facilitate more accurate structure–activity relationship predictions (Jumper et al., 2021). While ML and other bioinformatic approaches offer tremendous potential in characterizing gut MDPs, downstream validation involving rigorous in vitro and in vivo experiments is still critical and necessary. Initial research can rely on computational power, prioritize hits, and inform experiments, but wet lab work should complement it as often as possible.

In summary, the human gut microbiome has vast functional space and gut MDPs represent an exciting avenue of research and functional characterization. Various ML programs accept peptide sequence data and can predict bioactivity, and structural comparison tools allow for analysis beyond amino acid sequences. However, challenges exist that could limit the adoption and trust of ML programs in function prediction ability. Addressing these challenges and developing better, more accurate, and precise ML programs with corresponding tools for researchers will enhance the study of gut MDPs.

## AUTHOR CONTRIBUTIONS

**Ying‐Chiang Lee:** Conceptualization; writing—original draft; writing—review and editing.

## CONFLICT OF INTEREST STATEMENT

The authors declare no conflict of interest.

## ETHICS STATEMENT

None required.

## Data Availability

Not applicable.
